# Polyalanine Repeat Polymorphism in *RUNX2* Is Associated with Site-Specific Fracture in Post-Menopausal Females

**DOI:** 10.1371/journal.pone.0072740

**Published:** 2013-09-23

**Authors:** Nigel A. Morrison, Alexandre S. Stephens, Motomi Osato, Julie A. Pasco, Nicolette Fozzard, Gary S. Stein, Patsie Polly, Lyn R. Griffiths, Geoff C. Nicholson

**Affiliations:** 1 School of Medical Sciences, Griffith University, Gold Coast, Queensland, Australia; 2 Centre for Translational Medicine, Cancer Science Institute, National University of Singapore, Singapore, Singapore; 3 School of Medicine, Deakin University, Geelong, Victoria, Australia; 4 Vermont Cancer Center for Basic and Translational Research, University of Vermont, Burlington, Vermont, United States of America; 5 Department of Pathology and Inflammation and Infection Research Centre, School of Medical Sciences, Faculty of Medicine, University of New South Wales, Sydney, New South Wales, Australia; 6 Rural Clinical School, School of Medicine, The University of Queensland, Toowoomba, Queensland, Australia; The Children's Hospital of Philadelphia, United States of America

## Abstract

Runt related transcription factor 2 (RUNX2) is a key regulator of osteoblast differentiation. Several variations within the *RUNX2* gene have been found to be associated with significant changes in BMD, which is a major risk factor for fracture. In this study we report that an 18 bp deletion within the polyalanine tract (17A>11A) of *RUNX2* is significantly associated with fracture. Carriers of the 11A allele were found to be nearly twice as likely to have sustained fracture. Within the fracture category, there was a significant tendency of 11A carriers to present with fractures of distal radius and bones of intramembranous origin compared to bones of endochondral origin (p = 0.0001). In a population of random subjects, the 11A allele was associated with decreased levels of serum collagen cross links (CTx, p = 0.01), suggesting decreased bone turnover. The transactivation function of the 11A allele showed a minor quantitative decrease. Interestingly, we found no effect of the 11A allele on BMD at multiple skeletal sites. These findings suggest that the 11A allele is a biologically relevant polymorphism that influences serum CTx and confers enhanced fracture risk in a site-selective manner related to intramembranous bone ossification.

## Introduction

Increased fracture rates are found in patients with lower bone mineral density (BMD), although many fractures occur in patients who are not considered osteoporotic [1], [2]. Fracture risk is not fully explained by BMD as factors that are environmental and genetic are also involved [3]. Although the genetic architecture of fracture risk is complex and polygenic, a reasonable candidate gene is *RUNX2*: the protein RUNX2 is a critical regulatory factor in osteoblast and chondrocyte differentiation and essential for skeletal development [4]–[7]. *RUNX2* is expressed in mesenchymal progenitors and promotes hypertrophy of chondrocytes [4]–[7] and directs the differentiation of osteoblasts [8]. The combined functions of bone extracellular matrix formation and mineralization regulate bone volume and mineral density. RUNX2 associates with a beta subunit (Cbfβ) and forms transcriptional complexes to either activate or inhibit the transcription of target genes [9]. Such function transactivation assays have been used to correlate severity of *RUNX2* mutations with function [10].

Heterozygous mutations in coding and promoter regions of *RUNX2* in humans [4]–[7] cause the skeletal syndrome Cleidocranial Dysplasia (CCD) [11]. CCD develops when one allele of *RUNX2* is entirely functional and the other compromised, a state known as haploinsufficiency; indicating that RUNX2 levels are limiting in skeletal development. CCD is characterized by hypoplasia/aplasia of clavicles, persistently open or delayed closure of sutures, Wormian bones, supernumerary teeth, short stature and other skeletal abnormalities [11]. Certain bones are more affected than others in CCD: notably the clavicle, scapula, face/mandible, pelvis depending on the severity of the *RUNX2* mutation [10], [11]. During development, particular bones form by either endochondral or intramembranous ossification or a combination of both processes [12]. The bones most affected in CCD, such as scapula, face/mandible, pelvis and clavicle, have a high content of intramembranous ossification [12]. *Runx2* knock-out mouse homozygotes fail to develop osteoblasts and have no mineralized bone. In the case of haploinsufficiency in mice, greater defects are observed in bones with a higher content of intramembranous ossification compared to bones of greater endochondral origin, mirroring the subtly of human CCD. Regardless of the distinction between intramembranous and endochondral routes of formation, a feature of CCD is a characteristic range of bones that are affected in a particular rank order of severity. Studies of *Runx2* gene dosage using isoform specific knockout mice support the hypothesis that Runx2-II (an isoform driven off the P1 promoter) is more important for endochondral bone formation than the Runx2-I isoform (driven by the P2 promoter) [13]. To that point, mice with intact Runx2-I isoform survive with reduced bone density and are able to increase cortical bone over time, with a form of low bone turnover osteopenia [13]. However, mice with defects in the P1 promoter (which is induced in osteoblasts) that generate gene dose effects also mirror the defects in CCD with more pronounced defects in bones with a high content of intramembranous ossification [14]. Currently, these data taken together suggest that local tissue specific effects occur and that different ossification types (endochondral versus intra-membranous) are differentially affected by either RUNX2 isoform and/or *RUNX2* gene dosage. Based on these propositions, we present the hypothesis that genetic variants of RUNX2 with functional effects may manifest in relative differences of intramembranous bone versus endochondral bone. Although a number of genetic markers exist in *RUNX2* [4]–[7], we selected a coding region variant for study: a six amino acid deletion in a poly alanine tract that is a candidate functional polymorphism. Even if the distinction between intramembranous and endochondral bone ossification is not as great as supposed, functional variants of RUNX2 may present with a rank order of affected bones similar to the rank order of affected status found in CCD. However, if this hypothesis is correct, then different rates of fracture may be expected in bones of primarily endochondral origin relative to those of more intramembranous origin, according to *RUNX2* genotype.

## Materials and Methods

### Clinical collections

Geelong Osteoporosis Study (GOS): one component of the GOS comprises a prospective population-based epidemiology study with age-stratified random recruitment from the Barwon Statistical Division (population 216,000), as described previously [15], [16]. DNA, serum markers and bone densitometry DXA data were obtained for 822 females. Markers of bone turnover CTx (collagen telopeptide) and BSAP (bone alkaline phosphatase) were available on 803 and 799 subjects, respectively. DXA was performed using Lunar DPX-L densitometer and analyzed using Lunar DPX-L software version 1.31. BMD was measured at the spine (L2–L4) in the posterior-anterior projection, proximal femur (femoral neck, Ward's triangle, trochanter), whole body, ultradistal (UD) and mid-forearm sites. Bone mineral apparent density (BMAD) was calculated for the spine (L2-4) as BMC/(area)^1.5^ according to Carter *et al* [17]. Self-reported fracture history was obtained by questionnaire.

The GOS random population survey study subjects were recruited via the electoral roll and thus represent an unbiased random population survey. Follow up data were available: the cohort was followed prospectively and incident fractures occurring during the five year period following baseline identified from all radiological reports in the study region, using a validated keyword search strategy [18]. Furthermore, fractures resulting from high impact events such as motor vehicle accidents, were excluded. Within the postmenopausal females of the random population-based sample, a group existed (n = 224) that did not have incident fracture in the five year surveillance period and had reported an absence of adult fracture history at recruitment; representing a control non-fracture postmenopausal group with a mean age of 72±10 (± standard deviation, SD) years similar to that of GOS fracture cases (70±12 SD).

GOS fracture cases (GOS fracture): all residents of the Barwon Statistical Division who sustained incident fracture over a two year period were identified from radiology practices servicing the region, as previously described [19]. All fracture case were approached to join the study. DNA was taken from female fracture patients over 35 years of age with fractures of all types. In order to reduce confounding variables, this study is limited to post-menopausal females. A total of 598 fracture patients were genotyped. The International Classification of Diseases, 9th revision (ICD 9) was used to code fractures. For GOS and GOS fracture, written informed consent was obtained under the procedures of the Declaration of Helsinki, approved by the Barwon Health Human Research Ethics Committee.

South East Queensland bone study (SEQBS): the study is based at Southport in Queensland, Australia. SEQBS study participants were recruited via notices in the local newspaper, resulting in 980 volunteers. DNA was extracted from leukocytes using salt precipitation methods and genotype data obtained from 671 samples. For all subjects, anthropometric measures and a detailed history was taken of age of menarche, parity, menopausal status, smoking, and other history of illness. Fracture history was obtained through detailed questionnaire including minor or major severity of impact associated with injury, location of fracture and age at fracture. DXA data were not available. In addition, subjects had data on 23 standard serum and urine metabolic measures including alkaline phosphatase. Written informed consent was obtained and procedures were approved by the Griffith University Human Research Ethics Committee, following the guidelines of the Declaration of Helsinki.

### Serum measures of bone turnover

Serum from subjects of GOS and SEQBS were taken after overnight fast and stored at −80C. Enzyme-linked immunoassay was used to determine serum concentrations of cross-linked collagen products (CTx) and bone specific alkaline phosphatase (BSAP) [20], [21].

### Detection of alleles in *RUNX2* polyQ/polyA repeat sequence

Convention specifies that, for the human, the gene is termed *RUNX2* and the protein termed RUNX2. Primers were: forward 5′-AGC CTG CAG CCC GGC AAA ATG AGC-3′, reverse 5′-GGG TGG TCG GCG ATG ATC TCC ACC ATG-3′. PCR amplified DNA was resolved on 7.5% polyacrylamide enabling accurate detection of deletion genotypes as described [22]. All DNA samples were genotyped twice on two different occasions. Some DNA samples failed to amplify and therefore were not genotyped. There was no apparent pattern to those DNA that failed.

### Plasmids, cell culture and luciferase assay

Full length RUNX2 type-I cDNA (pEF-αA) [23] served as a template to construct RUNX2 11A allele cDNA. PCR was used to amplify the RUNX2 11A from a homozygous individual using the primers 5′-TTCACCACCGGACTCCAACT-3′ for the 5′ side and 5′-CATCTGGTACCTCTCCGAGGGCTACCACCTTGAAGGCCACGGGCAGGGTC-3′ for the 3′ side. The reverse primer contained an EcoNI tag facilitating the cloning of the PCR amplified product into the BglII-EcoNI site of pUC18RUNX2 [24]. The 11A RUNX2 cDNA was confirmed by DNA sequencing and was excised from pUC18 using XbaI restriction digest and cloned into the XbaI site of pEF-Bos. The human osteocalcin promoter vector pOSLUX (590 base pairs of the promoter upstream of fire-fly luciferase) was as described [24]. pRRE (meaning Runx-Responsive Element, and made for this study) contains three copies of the consensus RUNX2 binding element from the mouse osteocalcin gene upstream of firefly luciferase driven by a minimal promoter. Other constructs for glutamine variants 16Q and 30Q were described previously [24]. Cell lines were obtained from the American Type Culture Collection (ATCC). Transfection experiments and cell culture conditions were as described previously [24]. In summary, cell lines (NIH3T3 and HEK293) were cultured DMEM with 10% fetal bovine serum (v/v), 1% Penicillin-Streptomycin (v/v) in a 5% CO_2_ humidified atmosphere at 37°C. Transfections were done using FuGENE6 according to the manufacturer's protocol (Promega) as described previously [24] and luciferase assays according to Dyer et al [25]. Confocal microscopy was used to measure the extent of nuclear localization when transfected into COS7 cells; a cell line chosen due to low endogenous RUNX2 and ease of transfection. Cells were counted and categorized according to whether staining was nuclear, cytoplasmic or nuclear and cytoplasmic using a RUNX2 antibody.

### Statistical Analysis

Fisher's exact test and Pearson's chi-square test of association were used to analyze the allele frequencies between fracture and non-fracture groups. Probabilities (p values) are presented for the association of alleles (17A versus 11A) and the carrier status (11A/11A homozygotes considered equivalent to 17A/11A heterozygotes) using odds ratios (OR). In analysis of sub-categories of fracture, Fisher's exact test was used to provide the probability under the assumption that defective RUNX2 variants would predispose to greater defect (reflected in higher fracture rates) at bones of greater intramembranous content, as had been observed in mice [14]. Logistic regression was used to study the effects of covariates on binary outcomes. In the GOS randomly-selected sample, analysis of variance (ANOVA) was used to compare subject anthropomorphic measurements and the general linear model was used to compare BMD and biochemical markers of bone turnover between the different RUNX2 genotype groups while adjusting for the covariates age and weight. Fisher's protected least significant difference test was used for post-hoc comparisons, after a prior significant omnibus ANOVA F-test. Student's t-tests or ANOVA were used to analyse the quantitative transactivation analysis data. The general linear model was used for exploration of multifactorial relationships and to analyse transfection data with complex treatments. Equality of variance assumptions were met by logarithmic transformation, where necessary and verified using Levene's test. Fisher's method was used to combine p values in independent studies: (−2 times the sum of log_e_ p values is distributed as a chi-square with 2 k degrees of freedom, where k is the number of studies).

## Results

### Relationship of *RUNX2* 11A allele to risk of fracture GOS

RUNX2 alleles were observed at a frequency of 0.93, and 0.07 for 17A and 11A alleles, respectively. A significant difference was observed (p = 0.020, Table 1) in allele frequencies of 17A and 11A between the control no-fracture group with female fracture cases from the same region (the Barwon Statistical Division) with an increase of the 11A allele in the fracture group relative to the non-fracture group. The 11A allele frequency was 0.04 in the control non-fracture group and 0.07 in all fracture cases. Control subjects were recruited from the same population (GOS) and had not sustained fracture during the observation interval and had reported no history of adult fracture. When allele frequencies were compared between all fracture cases and controls, the OR for fracture related to the 11A allele was 1.9 with a confidence interval (95% CI) of 1.1 to 3.2.

**Table 1:** Study, group, genotype counts, 11A allele frequency (*f*[11A]) and p value using Pearson's chi square of allele counts in non-fracture control versus fracture cases in females from two studies (GOS and SEQBS) and those studies combined.

Study	Group	17A/17A	17A/11A	11A/11A	*f*[11A]	p value
GOS	Control	207	17	0	0.04	
	Fracture	518	78	2	0.07	0.020
SEQBS	Control	193	13	1	0.04	
	Fracture	71	14	1	0.09	0.005
Combined	Control	400	30	1	0.04	
	Fracture	589	92	3	0.07	0.0007

### Relationship of 11A allele to risk of fracture SEQBS study

In the South East Queensland Bone study (SEQBS), at a different geographical location within the same country, self reported history of fracture prior to the study was available for all genotyped subjects. The allele frequencies were 0.94 and 0.06 for 17A and 11A alleles respectively. There were no significant differences in height, age or weight between the fracture and non-fracture groups (males and females analyzed separately). RUNX2 11A carrier status (11A heterozygotes and 11A homozygotes) was associated with fracture in the entire group (p = 0.033, OR = 1.71, 95% CI = 1.04–2.83). This effect was driven by association in the females (p = 0.025, OR = 2.01, 95% CI = 1.08–3.75). The effect in females was driven by stronger association in the post-menopausal category (p = 0.005, OR = 2.91, 95% CI = 1.34–6.34) while in the smaller number of premenopausal females in the study (n = 90) there was no evidence of significant association with fracture.

In the GOS study, postmenopausal females from the random population sample who had not reported facture on questionnaire and who did not have fracture in a five year observation period were taken as no-fracture controls. Drawn from within the SEQBS, all female postmenopausal women who did not report fracture represent a similar control group. Genotype and allele counts were not significantly different (p = 0.14) from this SEQBS no fracture post-menopausal group to that of GOS no fracture controls, indicating in a second population that a control no fracture group has a similar distribution of alleles. Comparison between female controls and fracture cases showed significant association of 11A with fracture (Table 1, allelic association p = 0.005) with allele distributions similar to GOS. Although these two populations were recruited from geographically separate parts of the same country, the no fracture controls allele frequencies were not significantly different (p = 0.14): Table 1 shows p values for allelic association with fracture in the total data for Australian post-menopausal females (p = 0.0007). Fisher's method for combining p values of independent studies gave a similar outcome (p = 0.001).

### Relationship of *RUNX2* 11A allele to fracture at different bone sites

Data from both GOS and SEQBS showed similar association with fracture for the 11A allele. Fractures at distal radius, ankle, spine, arm and hip were available from SEQBS: 11A status was significantly related to distal radius (p = 0.002) and ankle (p = 0.01) fracture location, but not other skeletal sites. The GOS fracture study recruited a larger number of fractures and so has a greater capacity to discriminate fracture location. A significant increase in the frequency of 11A alleles was observed in distal radius fractures relative to control non fracture cases (p = 0.02). The GOS fracture group consisted of many fracture types (Table 2) with spine, distal radius, hip, arm and ankle being the most frequent. The frequency of the 11A allele within each particular fracture sub-group was investigated. Scapular and clavicle fractures were combined as fractures of the pectoral girdle. The allele frequencies for 11A were determined for each anatomical fracture site; and the 11A allele frequency ranged from zero to 0.25. When the 11A allele frequencies were ranked from highest to lowest (Table 2) the rank order of frequency of 11A alleles according to fracture site follows the sites of the skeleton most affected by the syndrome CCD. The rank order from highest to lowest for 11A allele frequency in fracture sub-groups was: face/mandible, ribs, clavicle/scapula, pelvis and metacarpals. These bones are also the most strongly affected in CCD, dependent on the severity of the particular mutation [11].

**Table 2:** Genotype counts, 11A allele frequency (*fr*[11A]) and p value according to anatomical locations ranked by the frequency of the 11A allele in the fracture group.

Fracture group	17A/17A	17A/11A	11A/11A	*fr*[11A]	p value^1^
All-fracture	518	78	2	0.07	
Patella	5	0	0	0.00	0.682
Upper leg	8	0	0	0.00	0.545
Foot	30	2	0	0.03	0.280
Hip	60	5	0	0.04	0.203
Toe	11	1	0	0.04	0.385
Spine	97	11	0	0.05	0.117
Finger/thumb	17	2	0	0.05	0.268
Carpals	7	1	0	0.06	0.351
Humerus	33	5	0	0.07	0.118
Forearm	42	5	1	0.07	0.067
Ankle	40	7	0	0.07	0.063
Distal radius	91	16	0	0.07	0.020*
Lower leg	35	7	0	0.08	0.044*
Metacarpals	7	2	0	0.11	0.132
Pelvis	13	4	0	0.12	0.041*
Ribs, sternum	16	5	1	0.16	0.003*
Scapula/clavicle	3	2	0	0.20	0.054
Face/mandible	3	3	0	0.25	0.011*

1 p value from Fisher's exact test.

* denotes nominally significant effect.

The GOS fracture cases were sorted according to the site of fracture and those bones related to the more extreme CCD symptoms were combined. Bones defined as “CCD-related” were: ribs, face/mandible, scapula, clavicle, pelvis, metacarpals, phalanges (n = 78) compared to all other fracture types (n = 520). Within all fracture cases there was a significant preference for fracture at CCD-related bones within 11A carriers (p = 0.002, OR = 2.3, 95% CI = 1.4 to 4.0), compared to fracture at non CCD-related bones. When fractures in bones thought to have a higher proportion of intra-membranous ossification were considered (scapula, clavicle, face/mandible, sternum and ribs), compared to all other fracture types, a significant association with 11A alleles was observed (p = 0.0001, OR = 3.0, 95% CI = 1.6 to 5.4). Compared to controls, the association was more prominent: 11A alleles were associated with fracture of bones derived primarily from intra-membranous ossification compared to non-fracture controls (p = 3.5×10^−6^, OR = 4.8, 95% CI = 2.3 to 9.9). For bones of primarily endochondral origin, two fracture types were available that provided sufficient numbers (108 and 65 for spine and hip fracture, respectively) to test the hypothesis that the 11A allele alters the liability for fracture of a bone according to the majority mode of ossification (either primarily intramembranous or endochondral). The 11A allele was significantly associated (p = 0.0001, OR = 3.9, 95% CI = 1.9 to 8.2) with fracture at bones of a greater content of intramembranous ossification compared with fracture of bones of higher endochondral ossification (spine and hip combined) (Table 3). Therefore, within fracture patients, 11A *RUNX2* allele carriers have a higher likelihood of fracture at certain CCD-related bones, of higher intramembranous ossification, compared to wild type 17A allele-carriers.

**Table 3:** RUNX2 11A allele is associated with fracture at bones with cleidocranial dysplasia-related bones of higher intramembranous ossification (pelvis, scapula, clavicle, ribs, sternum, face/mandible) compared with common fractures in bones of higher content of endochondral ossification (hip and spine combined).

Fracture type	17A	11A	*fr*[11A]	p value
Intramembranous	84	16	0.160	
Endochondral	330	16	0.048	0.0001

Allele counts, allele frequency (*fr*[11A]) and p value by Pearson's chi-square.

### Relationship of *RUNX2* 11A allele to CTx and BSAP markers

Markers of bone remodeling, serum CTx and BSAP, were available from the random population subjects recruited from the GOS. Carriers of the 11A allele had significantly lower ln(CTx) measures (5.47±0.91 SD, N = 91) compared to non-carriers (5.70±0.81 SD, N = 712, p = 0.018). When adjusted for age and weight, 11A carriers retained significantly lower CTx serum levels (p = 0.03). In contrast, there were no significant differences in ln(BSAP) levels between 11A carriers and non-carriers (p = 0.706). Adjusting for the significant covariates age and weight had no material effect on the result (p = 0.715). CTx was not available for SEQBS: BSAP was not related to 11A status in this study (p = 0.53).

### Biochemical analysis of 11A RUNX2 expression construct relative to 17A allele

**Figure 1:**
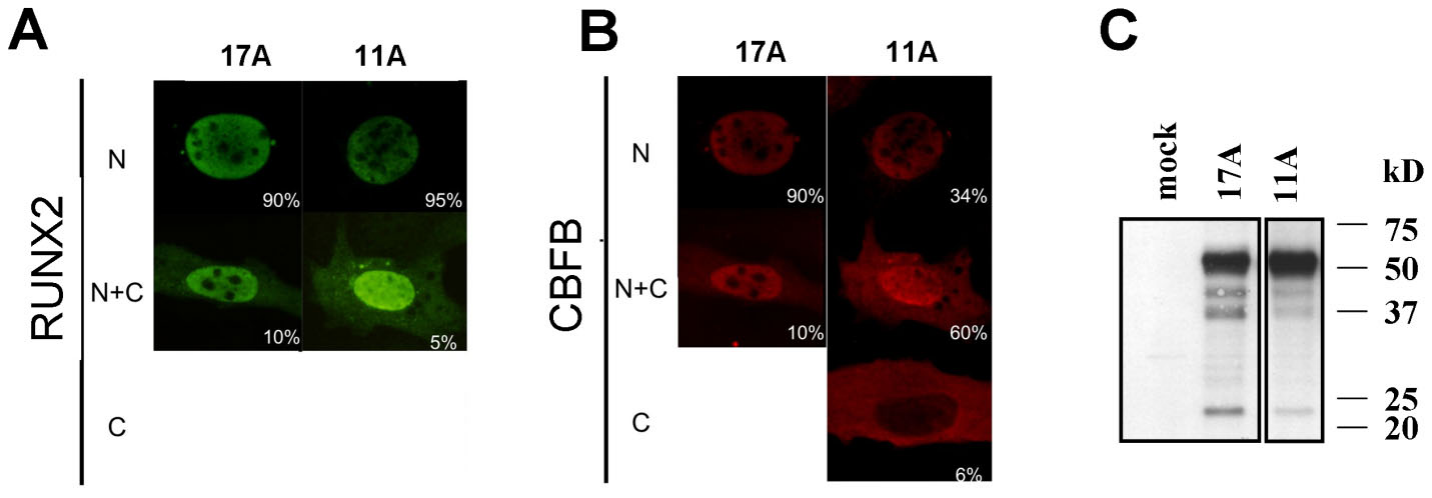
RUNX2 17A and 11A constructs have differential nuclear residence. A. Confocal immuno-fluorescence labeled with anti-RUNX2 antibody to detect nuclear or cytoplasmic residence. A comparison of RUNX2 17A and 11A reveals small differences in RUNX2 nuclear residence. B. the ability of RUNX2 to enhance the nuclear localization of the partner protein CBFB is diminished in RUNX2 11A isoform. White numbers indicate percentages of cells with that particular type of staining. C. Western blot indicating that expression vectors produce appropriate amounts of RUNX2 variants. Left and right panels are derived from the same gel, with intervening lanes removed; the left panel with mock transfected control and wild type 17A control appeared previously [24].

Expression vectors in the context of the RUNX2-I isoform were constructed containing either alternative allele of the alanine repeat (17A or 11A) and used in a series of biochemical tests to determine if significant differences existed between the isoforms. Under identical conditions of transfection there was no significant difference in the extent of nuclear localization of either isoform (p = 0.18), where the majority of staining was nuclear (Fig. 1A). However, under the condition of co-transfection of the co-activator protein CBFB, which forms a heterodimer with RUNX2, a significant difference (p = 1×10^−8^) was observed with less nuclear staining of CBFB in cells transfected with the 11A version of RUNX2 (Fig. 1B). In other words, a biochemical difference was only revealed by the presence of CBFB co-transfection, suggesting a reduced capacity to assist in CBFB nuclear localization. Western blots using extracts from COS7 cells transfected with expression vectors showed equivalent amounts of protein (Fig. 1C).

**Figure 2:**
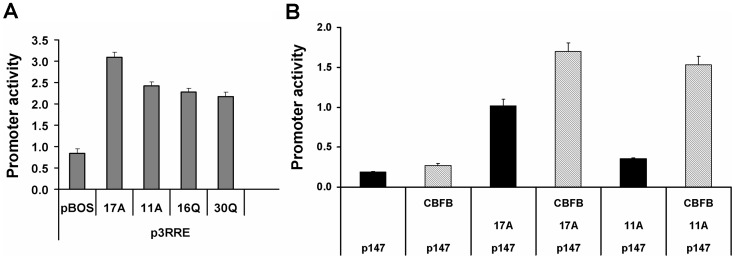
RUNX2 17A and 11A constructs have differential biochemical behavior. **A**. Transfection comparison of the activities of RUNX2 expression constructs driving RUNX2 sensitive reporter plasmid (p3RRE) in HEK293 cells. pBOS is empty vector control for baseline promoter activity of the RUNX2 responsive plasmids. 23Q/17A construct (17A) has higher activity than the RUNX2 23Q/11A variant allele (11A). On the reporter (p3RRE) the RUNX2 23Q/11A variant was significantly different from the wild type 23Q/17A allele (p = 0.0004) and was similar in expression to rare allelic variants of the glutamine repeat (16Q/17A and 30Q/17A, labeled as 16Q and 30Q, respectively). B. Effect of CBFB cotransfection on the comparison between RUNX2 17A and 11A constructs on a truncated mouse osteocalcin promoter, p147. A difference is observed between RUNX2 17A and 11A constructs in the absence of CBFB. In the presence of CBFB, the p147 target promoter activity is induced and the difference between RUNX2 17A and 11A constructs is eliminated.

Quantitative transactivation analysis was carried out using a RUNX2 target gene reporter assay. Based on power calculations, five constructs and controls were compared, with 9 replicate transfections done to detect minor effects. The HEK293 cell line was used due to low endogenous RUNX2. RUNX2 23Q/11A expression construct was compared with wildtype 23Q/17A and two glutamine repeat variants that we had already established were significantly different from the wild type (16Q/17A and 30Q/17A RUNX2 variants) [24]. As expected, the previously reported significant decrease in 16Q and 30Q rare RUNX2 variants was verified using reporter plasmids pRRE (Fig. 2A) with 16Q and 30Q constructs being 76% and 69% active. Similarly, compared to wildtype 23Q/17A, the 23Q/11A construct produced significantly lower activity (p = 0.0004), being 77% active overall in this assay.

Notably, a greater difference between RUNX2 17A and 11A isoforms was also observed on the truncated p147 mouse osteocalcin promoter, transfected into NIH3T3 fibroblast cells, where the RUNX2 11A isoform had lower transactivation capacity in the absence of CBFB (p = 0.001, Fig. 2B). Co-transfection of CBFB partner protein essentially normalized the effect to the level found in the 17A wild type isoform (p = 0.3). These data support the hypothesis that the 11A variant of RUNX2 is slightly decreased in potential transcriptional activation of target promoters and this effect varies according to the particular target promoter examined and the presence of cofactor proteins such as CBFB.

**Figure 3:**
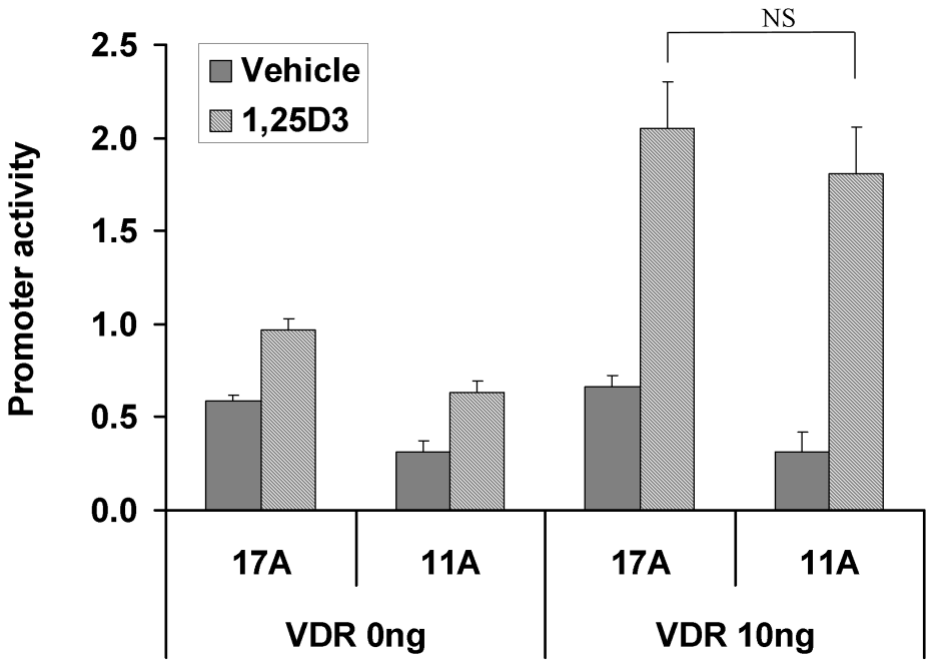
Effect of VDR transfection on difference between RUNX2 17A and 11A constructs in transfection. Constructs were transfected at four concentrations (1, 15, 30 and 45 ng) with or without VDR (10 ng) and with or without treatment with 1,25(OH)_2_D_3_ (1,25D3 in figure) at 10^−7^ M. Data presented are the marginal means derived from the general linear model and adjusted by linear regression to 12.5 ng of transfected vector. Error bars are one standard error of the mean. Promoter activity is the control *Renilla* adjusted pOSLUC firefly luciferase activity. In all cases within a treatment, is the comparison between RUNX2 17A and 11A significantly different excepting the single case marked where cells were transfected with VDR and treated with 1,25(OH)_2_D_3_.

Considering that CBFB appeared to modulate the effect of the RUNX2 11A allelic isoform in the expression construct relative to 17A, we explored the relationship by transfection with another partner protein of RUNX2, the vitamin D receptor (VDR) using the human osteocalcin promoter driving luciferase (pOSLUC) in HEK293 cells. This experiment compared simultaneously two constructs (RUNX2 17A and 11A) at three concentrations of transfected construct (control zero, 15, 30 and 45 ng transfected), with or without transfected VDR (10 ng) and with or without treatment with 1,25(OH)_2_D_3_ (at 10^−7^ M) with appropriate vehicle and empty vector controls, as duplicate transfections within each experiment [24]. This entire experimental plan was repeated and assayed on four separate occasions. No significant difference was observed in experimental repeats done at different times: data were pooled and analyzed using the general linear model taking into account all variables. VDR transfection did not have an effect on basal activity of the target pOSLUC in the absence of 1,25(OH)_2_D_3_: induction by 1,25(OH)_2_D_3_ in the presence of VDR was around three-fold. Treatment with 1,25(OH)_2_D_3_ in the absence of transfected VDR resulted in significant induction (1.6 fold, p = 3.9×10^−7^) presumably from endogenous VDR. The RUNX2 11A construct had significantly lower capacity to drive pOSLUC in HEK293 cells (p = 1.1×10^−6^). The difference between RUNX2 11A and 17A construct activity (Fig. 3) was significant in the: (i) absence of both VDR and 1,25(OH)_2_D_3_ (p = 0.01); (ii) absence of VDR and presence of 1,25(OH)_2_D_3_ (p = 0.001); (iii) presence of VDR but absence of 1,25(OH)_2_D_3_ (p = 0.013). A significant difference was not observed in the presence of VDR transfection and treatment with 1,25(OH)_2_D_3_ (p = 0.24), suggesting that activated VDR drives the promoter to an extent that covers any potential difference between RUNX2 17A and 11A. Because of the decreased basal activity (Fig. 3) of the RUNX2 11A construct driving pOSLUC there appears to be a greater response to 1,25(OH)_2_D_3_ than the wild type 17A construct (5.8 fold for 11A versus 3.1 fold for 17A). This means that in the presence of VDR and 1,25(OH)_2_D_3_ treatment, the difference between RUNX2 11A and 17A constructs is reduced and no longer significant since VDR induction occurs in the presence of both RUNX2 11A and 17A.

### BMD relationship

The effect of the alanine deletion polymorphism on BMD was investigated in the GOS random population subjects. BMD information was available from seven skeletal sites and a total of 822 subjects with genotype: all individuals were included in analysis. There were no significant differences in age, height and weight between the *RUNX2* genotype groups. The 11A allele did not show a significant relationship with BMD at any of the skeletal sites, including the distal forearm, in keeping with a prior publication on a smaller set of the same cohort [22].

## Discussion

To expand our prior studies of the GOS group [22], in this current work we doubled the sample of GOS random population subjects that were genotyped and completed the genotype of all available adjudicated fracture cases in the GOS fracture study. In addition, we show a relationship with self-reported fracture in a second independent cohort. In the GOS fracture cohort, we found a site-specific relationship of the 11A allele with fracture that was particular to bones with a greater content of intramembranous ossification. A relationship with BMD was not found, although a significant relationship with serum CTx in the GOS random cohort is evidence for a physiological relationship at the level of bone, since CTx is a marker of bone turnover [20]. A simple explanation may be that the effect of the 11A variant on BMD is small enough to escape detection in this study. The fact that we observed a relationship with fracture at the distal radius, but did not observe a relationship with BMD at this site suggests a non BMD-related mechanism should be considered. It is possible that the increased risk of fracture associated with the 11A allele is not explained by changes in BMD, but relates to the process of ossification and some unmeasured architectural parameter in bone. The long bones including the distal radius contain a bone collar where intramembranous ossification occurs and the periosteum is described as defective in CCD [11], [12]. Since the bone sites where the *RUNX2* 11A allele is most associated with fracture have a higher content of intramembranous ossification, the relationship of 11A to bone density and structure needs to be investigated in further studies, with that hypothesis in mind. Aspects of RUNX2 function in bone may influence an individual's susceptibility to fracture by altering components of bone quality that are independent of bone density as measured by DXA. While site selectivity was shown with reasonable numbers in two studies at the distal radius and ankle, a limitation of the study is that smaller numbers of fractures in other bones were available. Despite this, a simple classification system for the skeleton based on those bones most affected in CCD produced a statistically significant outcome, for increased fracture within bones of higher intramembranous content versus high endochondral content for carriers of the 11A allele.

There are limitations to this study. The fractures that comprise the majority of the study (GOS fracture) are adjudicated from medical records, but the second study (SEQBS) relied on self-reported history. Although we did detect significant alterations in RUNX2 activity, these were entirely *in vitro* assays in immortal cell lines and involve transfections with expression vectors and synthetic targets. There may be other target genes that show greater differential activity or indeed, no differential activity. Furthermore, this alteration in transactivation function may not be related to the fracture phenotype; it is possible that some other biochemical effect is occurring, specific to intramembranous ossification. Similarly, the BMD measures were mostly not in the bones that showed association with fracture. Although the association with fracture is based on reasonable numbers, the particular inferences that we draw concerning intramembranous bones are based on smaller numbers (although significant) and should be re-tested in large numbers. Finally, our study was biased towards post-menopausal females, because the fracture rate is higher in that group.

The 11A polymorphism is puzzling, being a protein coding variant of an important transcription factor that exists at reasonable frequency in the human population. Examination of primate sequences indicates that the 17A allele is conserved and presumably ancestral: Gorilla (22Q/17A) and chimpanzee (25Q/17A) are different from human in the glutamine repeat, while orangutan (23Q/17A) is identical to human. The Galago (bushbaby, *Otolemur crassicaudatus*) is evolutionarily very distant from humans, yet has 17 alanines (21Q/17A). Of 10 sequenced species available, the only primate RUNX2 that is not 17A is the mouse lemur (*Microcebus murinu*s) with 16A. This indicates remarkable stability of this sequence through time and reaffirms the peculiarity of the 11A allele, which is unique to humans. Given the conservation of the 17A configuration in the repeat over evolutionary time, the possible function of the 11A allele is intriguing. In the comparison between Neanderthal and modern human DNA sequences, *RUNX2* was indicated as highly subjected to genetic selective sweep, indicating evolutionary selection across this locus [26], [27]. *RUNX2* is therefore a candidate gene for differences between Neanderthal and modern human and a number of different functional changes may be expected within *RUNX2*. The available Neanderthal sequence is consistent with 17A in the repeat (see genome.ucsd.edu). Notable skeletal differences between Neanderthal and modern human include ribs and mandible, sites with high intramembranous content. Likewise, the available *RUNX2* sequence of another archaic human, the Denisovan hominin specimen [28], is consistent with the 17A allele (see genome.ucsc.edu). How does our data relate to these points? The *RUNX2* 11A allele may represent a rather new allelic variant, perhaps influencing intramembranous versus endochondral ossification, that is specific to modern humans.

The extent to which ossification at a particular bone is either endochondral or intramembranous in character may be an important factor in understanding the relationship of genes to bone and bone fracture as a trait. Association of *RUNX2* 11A allele with fracture exists in bones of greater intramembranous ossification content: so called intramembranous bones [29]. The same assemblage of bones is most affected in CCD, where RUNX2 levels are inadequate in certain tissues due to haploinsufficiency resulting in a critical threshold effect for abnormality where 70% activity or less in mouse caused CCD and greater than 79% activity resulted in normality [29], [30]. Therefore it seems likely that the mechanism of *RUNX2* 11A allele should relate to effective levels of RUNX2. This was supported by our transfection studies that showed RUNX2 11A was less effective at transcriptional activation through a transfection assay and had differential measures of nuclear binding.

An alternative explanation is that *RUNX2* 11A is not a causative variant but rather is in linkage disequilibrium with another unknown variant that is responsible for the molecular mechanism. Whole genome analysis shows association of BMD to the *RUNX2* genomic region [31]. Earlier studies indicated association of *RUNX2* alleles with bone phenotypes, BMD and fracture in a number of different populations [22], [24], [32]–[38]. We reported previously that the 11A allele arose in the common ancestral P2 promoter haplotype [32], and that a rarer haplotype variant of the P2 promoter with differential promoter activity was associated with higher BMD. This association was replicated in two separate Spanish cohorts [33], [34], Russians [35] and Koreans [36]. The P2 promoter drives RUNX2-I isoform, which has been associated with intramembranous ossification in mouse studies [39]–[41]. Differential activity of the *RUNX2* P2 promoter, changing the relative amount of the RUNX2-1 isoform might be an attractive hypothesis to explain differential fracture at bones of intramembranous ossification. Therefore, although our transfection data indicates that the 11A repeat polymorphism shows some functional differences, promoter polymorphism is still a candidate explanation of the association of 11A alleles with fracture: more studies are required on this topic. Other evidence of clinically relevant functional variation in *RUNX2* include association with bone length in hand and femur [35], [37]. Comparing other species for functional correlates of the Q/A repeat provides evidence that the Q/A repeat itself has function. Functional evidence for *RUNX2* Q/A repeat variation include association with skull shape specifically in dogs [42] and snout length more generally in *Carnivora* [43]. Although this effect was not generalized across all species of mammal [44] it was also significant in bill length within species of the family *Scolopacidae* shorebirds [45]. Furthermore, in this study we verified our prior transfection observations on RUNX2 glutamine repeat variants, which are infrequent and have a greater effect in transfection compared with the 11A variant. Taken together, these data suggest *RUNX2* as a locus of rich genetic variation related to skeletal evolution across species and a source of clinically relevant variation in humans.

In conclusion, the 11A allele of *RUNX2* is a frequent polymorphism (5% of alleles) related to increased risk of fracture. In particular, the 11A allele predisposes to fracture of those bones where a greater content of intramembranous ossification occurs. The 11A deletion allele had a significant effect on CTx in the GOS random population subjects but not BSAP. A direct effect of the deletion polymorphism on the transactivation ability of RUNX2 was observed. These findings suggest that the 11A deletion is a biologically significant polymorphism altering bone parameters and conferring an increased risk of fracture in a site selective manner. Further to this point, we suggest that future whole genome analysis of fracture take into account fracture subtypes based on categories of intramembranous bone ossification.
